# Characterization of YKL-40 Binding to Extracellular Matrix Glycosaminoglycans

**DOI:** 10.3390/md23100379

**Published:** 2025-09-26

**Authors:** Unnur Magnusdottir, Yiming Yang Jonatansdottir, Kristinn R. Oskarsson, Jens G. Hjorleifsson, Jon M. Einarsson, Finnbogi R. Thormodsson

**Affiliations:** 1 Genis hf, 580 Siglufjordur, Iceland; jon@genis.is; 2 School of Health, Business, and Natural Sciences, University of Akureyri, 600 Akureyri, Iceland; finnbogi@unak.is; 3 School of Engineering and Natural Sciences, Science Institute, Department of Biochemistry, University of Iceland, 102 Reykjavik, Iceland; yyj1@hi.is (Y.Y.J.); kro@hi.is (K.R.O.); jensgh@hi.is (J.G.H.)

**Keywords:** YKL-40, glycosaminoglycans (GAGs), extracellular matrix (ECM), binding affinity, Microscale Thermophoresis (MST), molecular docking, inflammation, fibrosis, cancer

## Abstract

YKL-40 is a chitinase-like glycoprotein implicated in various pathological processes, yet its glycosaminoglycan (GAG) binding profile beyond heparin has not been examined. In this study, we performed a Microscale Thermophoresis (MST) analysis on the heparin-binding glycoprotein YKL-40 using low molecular weight GAG oligosaccharides. We identified two new GAG ligands, dermatan sulfate (DS) and hyaluronan (HA), while chondroitin sulfate (CS) showed no detectable binding affinity. The results show that heparin is bound with the strongest affinity, followed by DS and HA. To further investigate these differences, molecular docking was used to evaluate possible binding modes. Molecular docking results indicated that both heparin and DS interacted with the same site on YKL-40, the heparin-binding site at residues 143–149, suggesting a multifunctional binding region that may act as a competitive switch or integration hub for spatially regulated signaling. Together, these findings expand the known ligand profile of YKL-40 and offer new insights into its ECM-context-dependent roles, with implications for targeting YKL-40 in diseases involving chronic inflammation, fibrosis, and cancer progression.

## 1. Introduction

YKL-40, also known as chitinase-3-like protein 1 (CHI3L1), is a secreted mammalian glycoprotein that belongs to the glycoside hydrolase family 18 (GH18). YKL-40 lacks enzymatic activity due to a mutation of Glu to Leu in the active site but it still binds chitin and chitooligosaccharides (ChOS) with micromolar affinity [1,2,3,4]. Chitin is a linear polysaccharide composed of β-(1→4)-linked N-acetylglucosamine (GlcNAc) units, forming a crystalline structure found in fungal cell walls and arthropod exoskeletons. Chitin is known to bind to the conserved chitin-binding site of the TIM barrel fold, characteristic of GH18 proteins [1,2]. Additionally, YKL-40 binds heparin/heparan sulfate [1,4,5,6,7], and the heparin-carrying receptors syndecan-1 [8], syndecan-4 [9], and CD44v3 [10]. Other known YKL-40 receptors are IL-13Rα2 [11,12] and TMEM219 [13]. While YKL-40’s ability to bind heparin is well established, the precise location of the heparin-binding site remains uncertain. Two separate regions have been proposed: a heparin-binding motif at residues 143–149, GRRDKQH [1,7], and a KR-rich region at residues 334–345 in the C-terminus [6,14]. The former site contains the typical heparin-binding motif BBXBXB (B is basic amino-acid, while X is any other) and has been suggested, by in silico studies, to bind heparin [7], while the latter, despite lacking this common motif, has been proposed as the functional binding site [6,14]. These regions have been explored via computational predictions and mutagenesis studies; however, a definitive structural confirmation by crystallization of the heparin-binding site location is still lacking [1].

YKL-40 is expressed by various cell types, including macrophages, neutrophils, fibroblasts, and cancer cells [15,16,17]. Its expression is upregulated by pro-inflammatory cytokines, such as interleukin-6 (IL-6), interleukin-1β (IL-1β), tumor necrosis factor-α (TNF-α), and transforming growth factor-beta (TGF-β) [15,18,19]. Upon binding to its receptors, YKL-40 activates intracellular signaling pathways, such as FAK, MAPK/ERK, and PI3K/AKT [20,21,22], which promote cell proliferation, migration, angiogenesis, and tissue remodeling [8,15,16,17,23,24,25]. Elevated levels of YKL-40 have been observed in numerous chronic inflammatory and fibrotic diseases characterized by extracellular matrix (ECM) dysregulation, such as idiopathic pulmonary fibrosis [13,26], asthma [27,28], and cancer [29,30,31]. In these conditions, YKL-40 correlates with disease progression, severity, and resistance to therapy [32,33,34]. Blocking YKL-40 has been shown to reduce fibrosis, modulate immune responses, and decrease cancer invasiveness [23,35]. Consequently, YKL-40 has been proposed both as a putative biomarker and a therapeutic target in these disorders. Preclinical studies using neutralizing anti-YKL-40 antibodies, siRNA, and other YKL-40-inhibitors are ongoing [14,15,16,17,35,36,37,38].

ECM remodeling is a hallmark of many chronic inflammatory and fibrotic diseases, where it plays a key role in regulating immune responses, angiogenesis, and tissue repair [39,40]. A central part of the ECM are the glycosaminoglycans (GAGs), which are linear, negatively charged polysaccharides composed of repeating disaccharide units. They are classified into sulfated (heparin/heparan sulfate [Hep/HS], chondroitin sulfate [CS], dermatan sulfate [DS], and keratan sulfate [KS]) and non-sulfated (hyaluronan [HA]) types. These GAGs vary in their degree of sulfation, site of sulfation within the sugar units, and backbone flexibility. HA contributes to ECM viscoelasticity, hydration, and scaffolding [41], while sulfated GAGs form proteoglycans (PGs) and act as signaling platforms by binding a range of proteins, including growth factors, chemokines, and cytokines [42,43,44]. Heparin, for example, is composed of GlcNS,6S-IdoA,2S, making it tri-sulfated. CS is primarily mono-sulfated, composed of GlcA-GlcNAc with sulfation typically at the 4- or 6-position of GalNAc. However, DS is derived from CS through the epimerization of glucuronic acid (GlcA) into iduronic acid (IdoA), resulting in a similar degree of sulfation but introducing greater flexibility and a different sulfate group orientation compared to CS [45,46]. These structural differences between the GAGs affect their protein-binding interactions and signaling outcomes [47].

Despite YKL-40’s established binding to heparin and heparin-carrying receptors, its specificity towards other GAGs, such as HA, CS, and DS, remains to be explored. Based on their structural similarities and the non-specific nature of their electrostatic interaction, it is plausible that these alternative ligands can bind to YKL-40 at the heparin-binding site [7]. One example of such alternative binding is the binding of heparin and DS to the HCII protein (heparin cofactor II), where both GAGs trigger thrombin inhibition [48]. Previous studies on YKL-40’s function have focused almost exclusively on heparin-binding without addressing other GAGs as potential ligands. Understanding whether other GAGs serve as functional ligands could expand our view of YKL-40’s mechanism of action in disease and help assess the outcome of targeting its heparin-binding site.

This study aims to determine whether YKL-40 binds to GAGs beyond heparin, by measuring the binding affinity to GAGs with varying structural and sulfation properties. We employed Microscale Thermophoresis (MST) to measure the binding dissociation constant (K_d_) of recombinant YKL-40 and well-defined GAG ligands (HA, Hep, CS, and DS). Additionally, molecular docking analysis was used to provide structural insights into the protein–ligand interactions. By integrating an in vitro binding assay and in silico modeling, this study provides new evidence of YKL-40’s broader GAG-binding profile and advances our understanding of how sulfation patterns and sugar composition can influence its interactions.

## 2. Results

### 2.1. Selecting Glycosaminoglycan Ligands for In Silico and In Vitro Binding Studies

To identify potential GAG ligands for YKL-40, we first performed a literature review focused on known or predicted YKL-40 ligands. YKL-40 has been shown to bind chitin, heparin, and several heparan-sulfate carrying proteoglycans (HS-PGs) in vitro. Additionally, in silico studies have predicted interactions with HA at the chitin-binding site and with CS at the heparin-binding site (residues 143–149). We analyzed the structural features of these known and predicted ligands, as well as their reported or predicted binding sites on YKL-40. Using these insights, we identified other GAGs with similar physicochemical properties, such as charge density, sulfation pattern, and backbone flexibility, and evaluated their likelihood of binding. Given the anionic nature of GAGs, we predicted the major types of interactions to be electrostatic and/or hydrogen bonding to the positively charged surface area on YKL-40, with a stronger binding potential predicted when combined with favorable features such as increased flexibility. An exception of this was HA, which was predicted to bind to the chitin-binding site of YKL-40 via GlcNAc (similarly to chitin).

Table 1 summarizes the resulting putative GAG ligands, detailing their major chemical components, predicted binding-site interactions (including known binding sites or the relevant protein surface area), and assessed the binding likelihood. The binding likelihood was determined using a combination of computational binding support and physiochemical properties.

### 2.2. YKL-40 Exhibits Selectivity in Binding Affinities Towards Various GAGs

Binding affinities between YKL-40 and various GAG ligands were assessed using MST binding assays. The GAG ligands were selected based on the quality of their characterization, defined degree of polymerization (DP), and defined disaccharide composition. Heparin DP6, a known YKL-40 ligand, was included as a positive control to validate the YKL-40–GAG binding at the heparin-binding site in the MST assay. Analysis of the MST dose–response binding curves showed that YKL-40 has a binding preference to certain physicochemical properties of the disaccharide units within the GAG ligands. Among the GAG ligands tested, YKL-40 exhibited the strongest binding to heparin DP12 (K_d_ = 119 ± 36 μM), followed by moderate affinity to heparin DP6 (K_d_ = 234 ± 133 μM). However, due to aggregation detected in the measurement by the MST instrument in samples at high heparin DP6 concentrations, full saturation in the dose–response titration was difficult to reach, preventing accurate determination of the K_d_ value. Heparin has a repeating disaccharide unit IdoA,2S-GlcNS,6S, which is highly sulfated, suggesting that the degree of sulfation, chain length, and increased flexibility of IdoA backbone unit contribute to increased YKL-40 binding. DS DP6 (type CS-B) showed moderate affinity (K_d_ = 269 ± 30 μM); however, no binding was detected to CS DP6 (a mixture of types CS-A and CS-C, with 4-sulfation > 6-sulfation) under our MST assay conditions (Supplemental Figure S2). Both CS and DS used in this study were primarily 4-sulfated on GalNAc but differed in their uronic acid composition (GlcA in CS, IdoA in DS). This structural difference further supports the contribution of IdoA to increased YKL-40 binding. The comparison of heparin DP6 (highly sulfated) and DS DP6 (moderately sulfated) indicates that the 3D conformation and spatial arrangement of negative charges are more important than overall charges. In summary, for sulfated GAGs, the results indicate that YKL-40 has a structural preference for ligands with a higher degree of sulfation, longer chain length, and the presence of IdoA for increased flexibility that enables the optimal spatial arrangement of charges required for binding.

**Figure 1:**
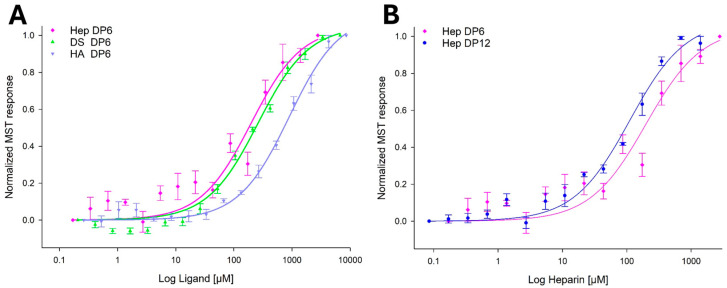
Comparison of the binding affinity curves between YKL-40 and GAG ligands measured by microscale thermophoresis (MST). (**A**) MST binding curves comparing YKL-40 binding to different GAG hexasaccharides (DP6). (**B**) MST binding curves showing size-dependent binding to heparin DP6 and DP12. An analysis of the dissociation constant (K_d_) was performed using His-tag labeled YKL-40 protein and four GAG ligands: heparin DP12 (Hep DP12, dark blue, *p* > 0.0001), heparin DP6 (Hep DP6, pink, *p* = 0.0037), dermatan sulfate DP6 (DS DP6, green, *p* > 0.0001), and hyaluronan DP6 (HA DP6, blue, *p* > 0.0001). Averaged data points were calculated from four MST binding curves and fitted with standard-error-of-mean (SEM) bars using nonlinear regression in SigmaPlot 13.0, with the Ligand Binding, one site saturation equation: f = Bmax × abs(x)/(K_d_ + abs(x)), with the parameter constraints Bmax > 0, and K_d_ > 0. The statistical significance of the K_d_ values was derived from the regression fit of the averaged data points, with Hep, DS, and HA showing significant binding, while chondroitin sulfate DP6 (CS) was not statistically significant (data shown in Supplemental Figure S2).

HA DP6 showed the weakest measurable affinity of the GAGs tested (K_d_ = 1010 ± 244 μM). HA is composed of GlcNAc and GlcA, where binding to the chitin-binding site may occur via GlcNAc, similarly to chitin, or at the heparin-binding site, through electrostatic and hydrogen bonding. Table 2 summarizes the binding affinity results for each GAG ligand, the major disaccharides of each GAG, and the observed binding affinity trend. Figure 1 shows the comparison of the mean fit between all ligands tested, and demonstrates that binding affinity increases with GAG chain length (Supplemental Figures S1 and S2 show all the ligand-binding curves for each GAG tested).

### 2.3. Molecular Docking Studies

A molecular docking analysis was carried out to evaluate the binding interactions between GAG ligands and YKL-40. Blind docking using AutoDock Vina identified the heparin-binding site at 143–149 as the preferred binding location for all three sulfated GAG ligands (heparin, DS, and CS). Next, a refined docking for enhanced binding accuracy was carried out (grid-box centered at this site using Glide). Two favorable binding poses were identified surrounding the residues R144 and R145 within the heparin-binding site at 143–149: Pose 1 (near the chitin-binding site) and Pose 2 (distal to chitin-binding site). For HA, both the chitin-binding site and heparin-binding site at 143–149 (Pose 1 and 2) showed stable binding; however, a better fit was observed to the chitin-binding site supported with salt bridges and H-bonds. However, some ligand poses generated included unfavorable proximity between the carboxyl groups of HA and Asp/Glu residues (<5 Å), suggesting unstable binding. A comparison between AutoDock Vina and Glide showed similar results with minor variations between the binding partners within the same binding location.

#### 2.3.1. Docking Analysis—YKL-40 and Chitin Tetramer

**Figure 2:**
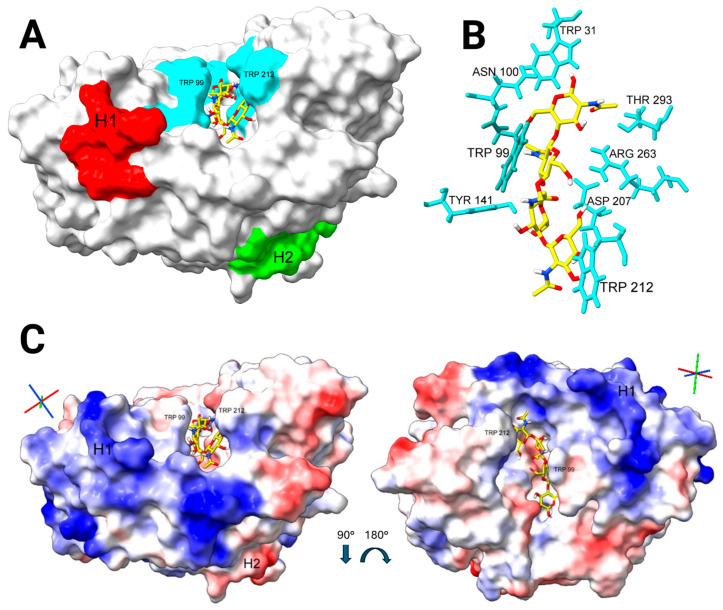
Surface representation of YKL-40 bound to a chitin tetramer (A4) in the chitin-binding site. (**A**) Surface representation of YKL-40 (PDB ID: 1HJW) and chitin tetramer (colored in yellow and using a heteroatom–stick representation) docking results. The two reported heparin-binding sites are highlighted: (H1) GRRDKQH at residues 143–149 (red), and (H2) the KR-rich site (green). Key interacting residues are shown in cyan, with Trp99 and Trp212 labeled to indicate the orientation of the protein. (**B**) Close-up of the docking results of the bound chitin tetramer (yellow) and key interacting residues in the chitin-binding site (cyan and labeled). (**C**) Electrostatic potential surface views (positive in blue, negative in red) of YKL-40 from two angles; to the left, oriented the same as A, and to the right, rotated 90° downward around the right-left horizontal axis (tilting forward), then 180° around the front-back axis (rotating 180° horizontally relative to the plane of the paper). The x (red), y (green), and z (blue) axes are included for visual reference of the protein’s orientation (Created in BioRender. Magnusdottir, U. (2025) https://BioRender.com/0ofg4jn).

Blind docking was carried out between YKL-40 and a chitin tetramer. Of the 10 poses generated, all bound within the chitin-binding site of YKL-40, with six top-ranked poses clustering to subsites −2 to +2, and four lower-ranked poses clustering to subsites −1 to +3 [1]. The most favorable fit was analyzed with no steric clashes and key interacting residues: Trp 31, Trp 99, Asn 100, Tyr 141, Asp 207, Trp 212, Arg 263, Glu 290, and Thr 293 (Figure 2). Compared to the crystallographic structure of YKL-40 in complex with chitin octamer (A8), the docking results were accurate for the key interactions between YKL-40 and the chitin oligosaccharide [2].

#### 2.3.2. Docking Analysis—YKL-40 and Heparin

**Figure 3:**
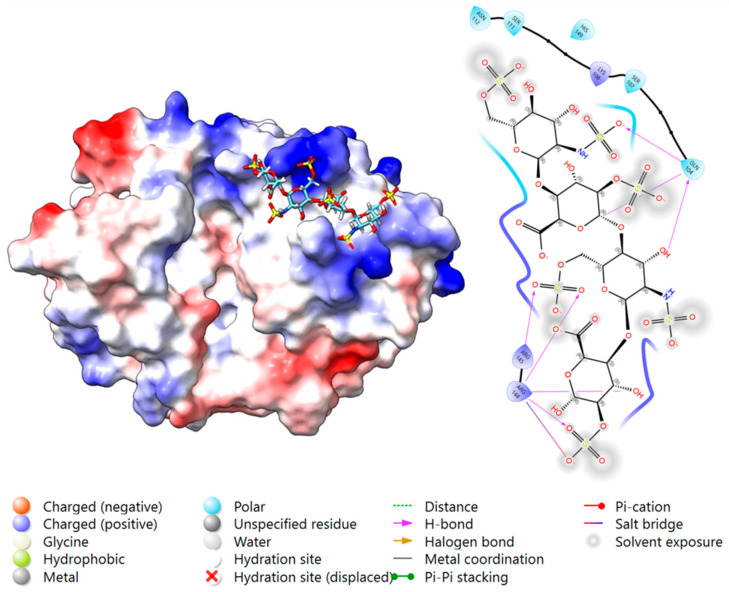
The predicted binding of heparin DP4 ligand to the heparin-binding site (H1) of YKL-40, highlighting the key interacting residues and bond types. To the left, a surface representation of YKL-40 (PDB ID: 1NWR) colored by electrostatic potential (positive in blue, negative in red), with the docked heparin DP4 ligand shown in cyan and using a heteroatom–stick representation. To the right, a 2D Ligand-Interaction Diagram (LID) of the docked heparin DP4 ligand with YKL-40, generated by Maestro, showing the predicted key interactions and bond type (Created in BioRender. Magnusdottir, U. (2025) https://BioRender.com/d43ahe5).

Blind docking of heparin DP4 with YKL-40 showed all 10 poses binding to the chitin-binding site, with some poses oriented towards the heparin-binding site at 143–149. However, the binding at this site was considered unstable due to repulsive forces between the sulfate-/carboxyl groups of heparin and carboxyl groups of Asp/Glu residues within the chitin-binding site. The blind docking was repeated with an occupied chitin-binding site (including the bound A4 ligand), which resulted in five poses near the heparin-binding site at 143–149. Refined docking at this site confirmed that Pose 1 binds with a salt bridge, H-bonding, and no steric clashes. The key interacting residues were: Arg 144, Arg 145, Gln 104, Lys 108, Ser 107, Ser 111, and His 149 (Figure 3, Supplemental Table S1).

#### 2.3.3. Docking Analysis—YKL-40 and DS

**Figure 4:**
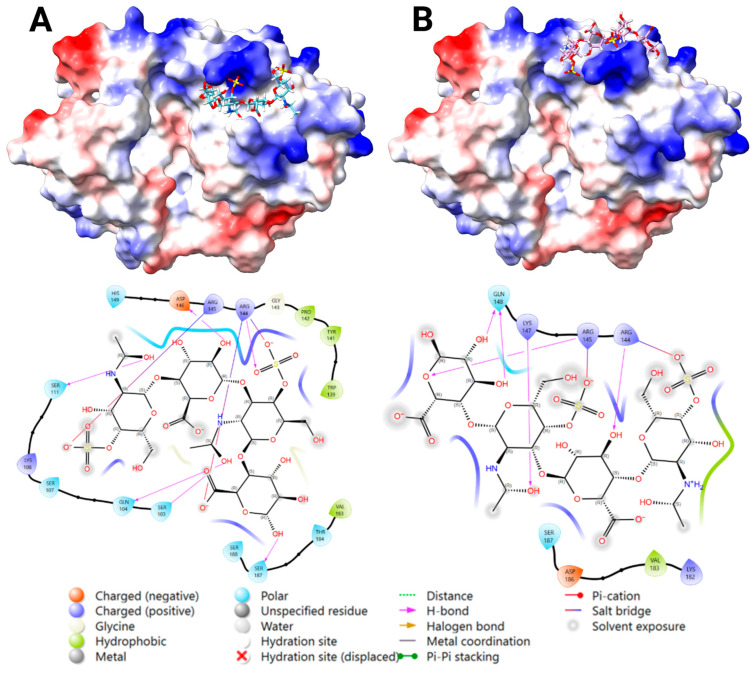
The two predicted binding locations of the dermatan sulfate (DS) DP4 ligand to the heparin-binding site (H1) of YKL-40, highlighting the key interacting residues and bond types. A surface representation of YKL-40 (PDB ID: 1NWR) colored by electrostatic potential (positive in blue, negative in red), with the docked DS DP4 ligand shown in cyan ((**A**): Pose 1), and pink ((**B**): Pose 2), and by a heteroatom–stick representation. A 2D Ligand-Interaction Diagram (LID), generated by Maestro, is shown below the protein structure, illustrating the predicted interactions between the ligand and the surrounding YKL-40 residues (Created in BioRender. Magnusdottir, U. (2025) https://BioRender.com/jt9z35a).

Blind docking of DS DP4 with YKL-40 generated 10 poses: five at the chitin-binding site, and five bound transversely bridging the chitin cleft reaching towards the heparin-binding site at 143–149. For the same reason as for heparin, blind docking was repeated with an occupied chitin-binding site, resulting in favorable binding for Poses 1 and 2, surrounding R144 and 145 within the heparin-binding site at 143–149. Centered docking at this site confirmed both binding poses, where the most favorable fit at each location showed binding via salt bridges and H-bonding, and no steric clashes (Figure 4). The key interacting residues were: Pose 1) Arg 144, Arg 145, Gln 104, Lys 108, Ser 103, Ser 111, and Ser 187; and Pose 2) Arg 144, Arg 145, Gln 148, Lys 147, and Lys 187. According to the docking score results, Pose 1 was significantly stronger than Pose 2 (Supplemental Table S1).

#### 2.3.4. Docking Analysis—YKL-40 and CS

**Figure 5:**
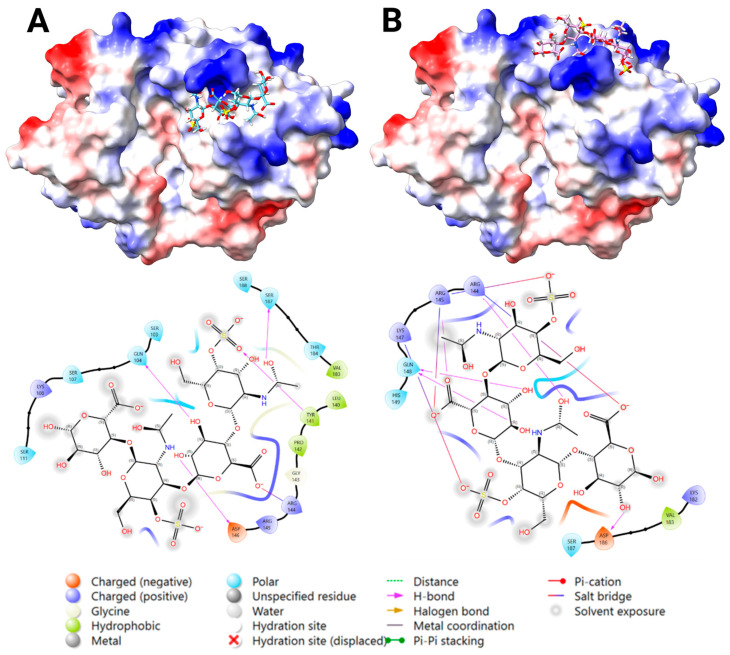
The two predicted binding locations of the chondroitin sulfate (CS) DP4 ligand to the heparin-binding site (H1) of YKL-40, highlighting the key interacting residues and bond types. A surface representation of YKL-40 (PDB ID: 1NWR) colored by electrostatic potential (positive in blue, negative in red), with the docked CS DP4 ligand shown in cyan ((**A**): Pose 1), and pink ((**B**): Pose 2) and by a heteroatom–stick representation. A 2D Ligand-Interaction Diagram (LID), generated by Maestro, is shown below the protein structure, illustrating the predicted interactions between the ligand and the surrounding YKL-40 residues (Created in BioRender. Magnusdottir, U. (2025) https://BioRender.com/d0i7co5).

Blind docking of CS DP4 with YKL-40 showed similar results as for DS docking, with some poses bound to the chitin-binding site, while most bound transversely bridging the chitin cleft reaching towards the heparin-binding site at 143–149. Blind docking was repeated with an occupied chitin-binding site, resulting in favorable binding for Poses 1 and 2 (Figure 5). However, centered docking at this site confirmed a favorable binding for Pose 2, whereas Pose 1 showed weak predicted affinity (Supplemental Table S1). The most favorable fit at each location showed binding via salt bridges, H-bonding, and no steric clashes (Figure 5). The key interacting residues were: (1) Arg 144, Gln 104, Ser 103, Ser 187, and Tyr 141; and (2) Arg 144, Arg 145, Gln 148, Lys 147, and Asp 186. The low binding score predicted for Pose 1 can be explained by the few interaction bonds depicted in the 2D ligand-interaction diagram (Figure 5), where sulfate- and carboxyl groups are not in a favorable position to form salt bridges with the key basic residues, compared to Pose 2.

#### 2.3.5. Docking Analysis—YKL-40 and HA

HA was predicted to bind the chitin-binding site of YKL-40, and thus, docking was performed on both the native YKL-40 and the apo form of the YKL-40–A8 complex (PDB ID: 1HJW, with A8 removed from the structure). Blind docking of HA DP4 with YKL-40 generated 10 poses: five at the chitin-binding site and five bound transversely to the chitin-binding site reaching the heparin-binding site 143–149, similar to the sulfated GAGs. Subsequently, centered docking was carried out on both the chitin-binding site (PDB ID: 1NWR and PDB ID: 1HJW with A8 removed from the structure) and the heparin-binding site at 143–149.

**Figure 6:**
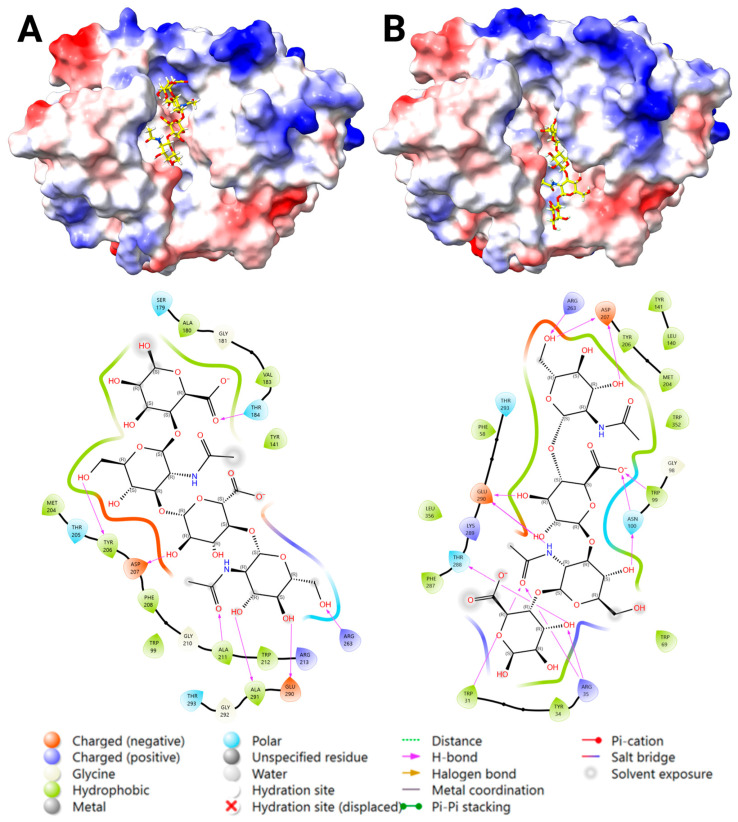
The two predicted binding poses of the hyaluronan (HA) ligand to the chitin-binding site of YKL-40, highlighting the key interacting residues and bond types. An analysis of HA DP4 docked to the chitin-binding site of YKL-40 ((**A**): PDB ID: 1NWR, ((**B**): PDB ID: 1HJW with A8 removed from structure). A surface representation of YKL-40 colored by electrostatic potential (positive in blue, negative in red), with the docked hyaluronan DP4 ligand shown in yellow and using a heteroatom–stick representation. A 2D Ligand-Interaction Diagram (LID), generated by Maestro, is shown below the protein structure, illustrating the predicted interactions between the ligand and the surrounding YKL-40 residues (Created in BioRender. Magnusdottir, U. (2025) https://BioRender.com/1mq2a3e).

Centered docking to the chitin-binding site generated 12 poses within the chitin-binding site (near −2 to +2 subsites, Figure 6A). The most favorable pose was analyzed with the key interacting residues Arg 263, Trp 212, Tyr 206, Ala 291, Thr 184, Asp 207, and Glu 290. However, some poses exhibited unfavorable proximity between carboxyl groups of HA and Asp/Glu residues (<5 Å), suggesting unstable binding. For centered docking to PDB ID: 1HJW, 12 poses were generated (near subsites −1 to −5, Figure 6B) with the same unfavorable repulsion. The most favorable pose was analyzed with the key interacting residues Arg 263, Arg 35, Trp 31, Trp 99, Asp 207, Asn 100, and Thr 288. A comparison of the docking results between PDB ID: 1NWR and PDB ID: 1HJW showed more favorable binding to PDB ID: 1HJW, suggesting that chitin-induced conformational changes within the crystal structure also favor hyaluronan binding (Supplemental Table S1).

**Figure 7:**
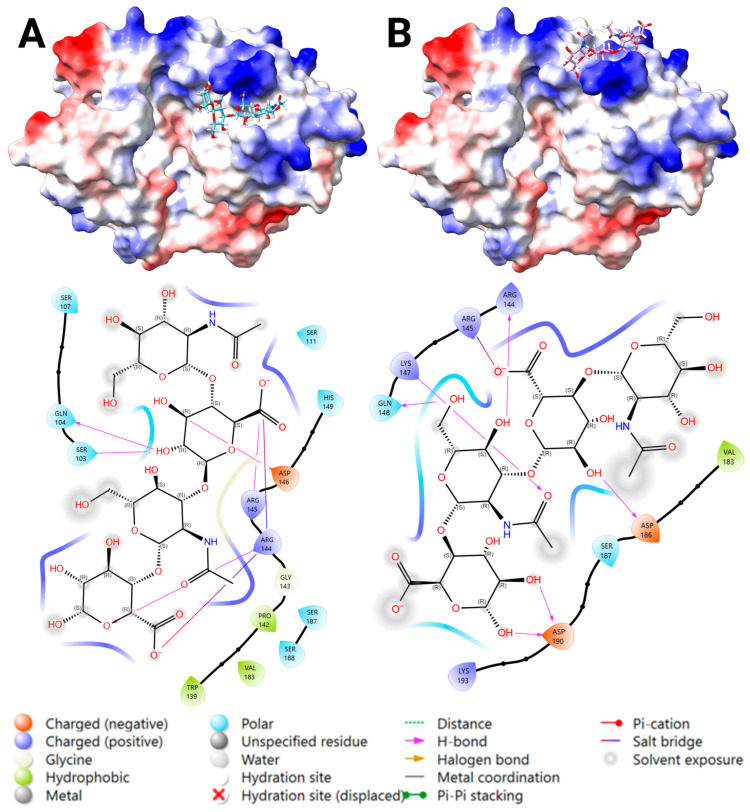
The two predicted binding locations of the hyaluronan (HA) ligand to the heparin-binding site (H1) of YKL-40, highlighting the key interacting residues and bond-types. An analysis of HA DP4 docked to the heparin-binding site at 143–149 of YKL-40 (PDB ID: 1NWR). A surface representation of YKL-40 colored by electrostatic potential (positive in blue, negative in red), with the docked HA DP4 ligand shown in cyan ((**A**): Pose 1), pink ((**B**): Pose 2), and using a heteroatom–stick representation. A 2D Ligand-Interaction Diagram (LID), generated by Maestro, is shown below the protein structure, illustrating the predicted interactions between the ligand and the surrounding YKL-40 residues (Created in BioRender. Magnusdottir, U. (2025) https://BioRender.com/9khue6d).

Centered docking to the heparin-binding site at 143–149 generated four poses, resulting in two favorable binding Poses 1 and 2, similar to DS and CS (Figure 7). The most favorable fit to each location showed binding via salt bridges and/or H-bonding and no steric clashes. The key interacting residues were: (1) Arg 144, Arg 145, Gln 104, Ser 103, and Asp 146; and (2) Arg 144, Gln 148, Ser 187, Lys 147, Lys 193, Val 183, Asp 186, and Asp 190. The glide docking score results showed more favorable binding for Pose 1 than Pose 2 (Supplemental Table S1).

## 3. Discussion

In this study, we investigated whether YKL-40 interacts with GAGs in the ECM beyond heparin. We identified two new YKL-40 ligands, demonstrating that it selectively binds not only to heparin, but also to dermatan sulfate (DS) and hyaluronan (HA), while showing no affinity for chondroitin sulfate (CS) under our experimental conditions. This selectivity highlights a binding preference for distinct structural properties, namely the degree of sulfation and the presence of IdoA in the GAG sugar backbone. The ligand selectivity was revealed by the binding hierarchy heparin > DS > HA. Notably, our in silico results showed that heparin and DS bind to the same site on YKL-40, suggesting it may serve multiple functions. Blind docking further revealed that sulfated GAGs preferentially interacted with the heparin-binding site at residues 143–149, but not with the KR-rich region near the C-terminus [6], consistent with our previous findings that chitin-binding allosterically affects this binding site [4]. This shared binding site could enable a competitive binding between structurally distinct GAG ligands, acting as a regulatory switch for YKL-40 function [50,51]. Alternatively, it may function as a convergent activation site. A well-characterized example of this is the protein HCII (heparin cofactor II), which binds both heparin and DS at the same site to activate thrombin inhibition and promote anticoagulant activity [48]. Given that heparin/HS and DS are distributed differently across tissues and cellular compartments, binding to this shared site may reflect a spatially adaptive mechanism, allowing YKL-40 to exert context-specific functions based on its surrounding microenvironment. This suggests a dynamic interplay within the ECM, where the relative abundance and composition of GAG ligands regulates YKL-40’s function.

MST assays confirmed that heparin has the highest affinity to YKL-40 of the GAGs tested. Heparin DP12 exhibited approximately twice the affinity compared to DP6 (K_d_ = 119 ± 36 μM and K_d_ = 234 ± 133 μM, respectively), likely due to the increased chain length enabling multivalent interactions. Crystallization of the YKL-40-heparin complex has proven challenging, likely due to the ionic nature of the binding and limitations posed by high-salt crystallization conditions [1]. Thus, we used molecular docking (AutoDock Vina and Glide Schrödinger) to gain structural insights into the protein–ligand interactions. Our docking results consistently revealed two favorable ligand poses for the sulfated GAGs near the heparin-binding motif at residues 143–149: Pose 1 is located adjacent to this motif (residues 143–149) but oriented closer to the chitin-binding site, and Pose 2 is located at the opposing side to Pose 1 (distal to the chitin-binding site). Both ligand poses are positioned in very close proximity and share key interacting residues, including R144, R145, and H149. Interestingly, heparin and DS preferentially bound to Pose 1 with interactions that extended the typical BBXBXB motif, expanding our understanding of the heparin-binding interface.

YKL-40 binding to heparin and heparan sulfate has been demonstrated in numerous experimental studies. Interaction with heparan sulfate (HS)-carrying receptors, such as syndecans and CD44v3, has been shown to play a significant role in cell migration, angiogenesis, and cancer progression [8,9,10]. Although heparin is primarily stored intracellularly within mast cell granules, it can be transiently released into the extracellular matrix during mast cell degranulation in response to pro-inflammatory stimuli [52,53]. Similarly, YKL-40 is upregulated during inflammation, suggesting that these two molecules could, in theory, be present in the same microenvironment during certain pathological conditions, potentially allowing for functional interactions.

YKL-40 bound DS with moderate affinity (K_d_ = 269 ± 30 μM), but surprisingly, no binding was detected between YKL-40 and CS under our MST experimental conditions, despite its structural similarity to DS. These results highlight the importance of IdoA’s added flexibility in positioning the sulfate groups adequately for interaction with the YKL-40’s heparin-binding site. Our docking results identified Pose 1 as a more favorable binding to DS, while Pose 2 was a better fit for CS. Interestingly, for CS, Pose 1 did not result in any salt bridge or hydrogen-bond formation between the sulfate groups of GlcA, but rather formed salt bridges with the carboxyl groups of the GalNAc units, resulting in poor overall binding and thereby supporting our experimental findings. This contrasts with the observed interaction with DS, where salt bridges and hydrogen bonds were formed between YKL-40’s basic residues and both the sulfate and carboxyl groups of DS, in addition to other supportive binding to the carboxyl and hydroxyl groups. This adds to the importance of IdoA’s enhanced flexibility, indicating an unfavorable position of the functional groups within CS that does not apply to DS. Combining our in vitro results with our in silico analysis, we propose Pose 1 (Figure 4A) as the functional heparin-binding site.

In our experimental setup, we used well-defined oligosaccharides to estimate YKL-40’s interactions with specific disaccharide subunits to identify the sequences to which it has the highest affinity. However, full-sized CS and DS in vivo are structurally heterogeneous polysaccharides, where CS can contain small amounts of IdoA and DS may include GlcA, making them more complex than the simplified oligosaccharides used here. Thus, while no binding was observed in our experiment for CS DP6 (GlcA-GlcNAc,4S > 6S), interaction with native CS in vivo cannot be ruled out. YKL-40’s selective binding to DS over CS may be relevant to pathological processes, such as fibrosis, cancer progression, and tumor angiogenesis, where both DS and YKL-40 play established roles [49,52,53]. In fibrosis, DS regulates the fibroblast growth factor (FGF) and platelet-derived growth factor (PDGF) availability and supports collagen I fibril formation by increasing the number and width of the fibers [43,54,55,56,57,58]. YKL-40 has been shown to increase the rate of collagen I fibril formation and to reduce MMP-1 activity [59]. Collagen I is also a known YKL-40 ligand. Biggs et al. identified three functional YKL-40 isoforms (cartilage major, cartilage minor, and chondrocyte-derived) that modulated the collagen I fibril formation differently [60]. Interestingly, the chondrocyte-derived isoform promoted fibrillogenesis, whereas the cartilage major isoform inhibited it [60]. These opposing effects suggest that YKL-40’s function is influenced by its surrounding ECM context during connective tissue remodeling.

In cancer, DS is overproduced and structurally remodeled to support invasive cell behavior. Elevated IdoA content in tumor-associated DS enhances hepatocyte growth factor (HGF)-mediated signaling and ERK1/2 activation [61,62], a pathway that YKL-40 is also known to modulate [63,64,65]. Moreover, both molecules contribute to angiogenesis: DS stimulates endothelial proliferation and MMP activity [66], while YKL-40 modifies MMP activity and activates endothelial cells and vascular smooth muscle cell migration via MAPK and Akt pathways [8,23,24,63]. Since YKL-40 and DS often appear in the same tissue environments and are linked to similar pathological conditions, this suggests a potential functional interaction. Based on our binding data and the literature, their activity might be the result of a direct binding interaction between YKL-40 and DS, resulting in profibrotic and cancer progression signaling. Further research is needed to determine whether specific DS-containing proteoglycans (PGs) could act as functional receptors for YKL-40, similarly to the HS-PGs syndecan-1 and syndecan-4 [8,9].

HA, a linear non-sulfated saccharide composed of GlcNAc and GlcA, showed low binding affinity in vitro (K_d_ = 1010 ± 244 μM), despite docking predictions suggesting strong binding to the chitin-binding site, as seen from our docking results and the literature [7]. This discrepancy likely reflects the limitations caused by rigid protein docking, which allows little-to-no protein flexibility (only minimal side-chain movements) and fails to capture dynamic interactions. Our post-docking analysis (summarized in Supplementary Table S1) revealed an electrostatic repulsion between the negatively charged carboxyl groups of HA (GlcA) and the acidic residues (Asp/Glu) within the chitin-binding site, which may contribute to an overestimation of the apparent binding affinity in silico compared to in vitro results. Unlike chitin and ChOS, HA contains GlcA that carries a negative charge, leading to this repulsion. The previously discussed role of IdoA′s flexibility and sulfate exposure does not apply to HA, which lacks both features. Nevertheless, HA may interact with YKL-40 at the heparin-binding site. Although CS has a higher overall negative charge, it showed no binding in vitro and exhibited lower predicted affinity in silico to the heparin-binding site, compared to HA (Supplemental Table S1). This suggests that HA′s potential binding to the heparin-binding site is not driven by the overall charge density, but rather by favorable 3D conformation and spatial arrangement of the charges. Due to HA’s weak binding in vitro and mismatching docking results at both chitin- and heparin-binding sites, its binding site on YKL-40 remains unclear. However, blind docking consistently placed HA at either the chitin-binding site or Pose 1 of the heparin-binding site, suggesting it may bind to one or both regions. While in silico predictions offer valuable insights into predicted protein–ligand interactions, our findings highlight the need for further research using complementary methods, such as X-ray crystallography, SAXS (Small-Angle X-ray Scattering), and/or NMR, to confirm the location of the heparin- and hyaluronan-binding sites.

Despite its weak affinity, the HA–YKL-40 interaction may still play a vital biological role in vivo [67]. Studies of HA’s interaction with CD44 show that even low-affinity, transient interactions can regulate key cellular processes, such as adhesion, migration, and signaling. This is largely due to their dynamic and reversible nature, making them suitable for rapid on/off interactions during processes like wound healing and tumor metastasis [68]. Likewise, Vaynberg et al. reported that weak binding events are not only common but essential for the regulation of reversible cellular processes, such as focal adhesion and signal transduction [69]. In this context, YKL-40’s HA binding may facilitate transient retention or competition with other ligands in the ECM, functions that are important in HA-rich environments, such as inflamed or remodeling tissues.

In conclusion, in this study, we identified DS and HA as new GAG ligands of YKL-40, revealing a selective binding beyond heparin. YKL-40 showed a clear preference for GAGs with a higher degree of sulfation and the presence of IdoA, as reflected in the binding hierarchy heparin > DS > HA, and no detectable binding to CS. Our in silico results indicated that both heparin and DS bind to the heparin-binding site at residues 143–149 on YKL-40, suggesting that this site may act as a convergent activation site or as an ECM-context-specific regulatory switch for YKL-40’s function. Despite the low binding affinity in vitro, HA’s interaction with YKL-40 may still support transient and reversible roles in HA-rich environments, such as inflamed and/or remodeling tissues. These weak yet functionally important interactions are consistent with the behavior of other HA-binding proteins, such as CD44. By identifying DS and HA as novel ligands of YKL-40, our findings significantly expand our current knowledge of YKL-40’s interaction profile within the ECM. This work provides a foundation for exploring YKL-40’s ligand-selective signaling mechanisms and opens new avenues for targeting its activity in disease-specific ECM contexts, such as chronic inflammation, fibrosis, and cancer.

## 4. Materials and Methods

### 4.1. Materials

The human embryonic kidney cell line 293T (CRL-3216™) was obtained from ATCC (American Type Culture Collection), Manassas, VA, USA. The plasmid preparation was purchased from GeneScript, the Netherlands (the final construct was CHI3L1_pcDNA3.1/Hygro(+)-C-6His, ORF clone NM_001276.3). The transfection reagent Turbofect™ was obtained from Thermo Scientific, Waltham, MA, USA. Cell culture medium and supplements were obtained from Gibco^®^ Life Technologies (Carlsbad, CA, USA). The Gibco™ Hygromycin B antibiotics and Nunc^®^ cell culture flasks were obtained from Thermo Scientific, Waltham, MA, USA. HisTrap™ HP 5 mL column was obtained from Cytiva, Marlborough, MA, USA. The HiTrap Heparin HP column was obtained from GE Healthcare Life Sciences, Chicago, IL, USA. A human YKL-40 ELISA kit was purchased from Cyclex Co., Nagano, Japan. A Stericup ultrafiltration unit (0.45 µm) and an Amicon ultracentrifugation device (10 kDa) were obtained from Merck Millipore, Burlington, MA, USA. The chitin oligosaccharide hexa-N-acetylchitohexaose (GlcNAc6, A6) was purchased from Isosep, Tullinge, Sweden. The GAG oligosaccharides heparin degree of polymerization (DP) 6 and DP12 (Cat. no. HO06 and HO12), chondroitin sulfate DP6 AC (Cat. no. CSO06), dermatan sulfate DP6 (Cat. no. DSO06), and hyaluronan DP6 (Cat. no. HA06) were purchased from Iduron, Manchester, UK. Monolith standard capillaries and His-Tag Labeling Kit RED-tris-NTA 2nd Generation were purchased from NanoTemper Technologies GmbH, Munich, Germany.

### 4.2. Transfection and Production of YKL-40

The human YKL-40 (NM_001276.3) cDNA sequence was cloned into the plasmid vector pcDNA3.1/Hygro(+)-C-6His (C-terminal 6x histidine tag and hygromycin resistance gene) by GeneScript. HEK293T cells were transfected with the YKL-40 plasmid using a Turbofect™ transfection reagent, following the manufacturer’s instructions. Following the incubation of HEK293T with a YKL-40 plasmid for 48 h, transfected cells were selected using a 400 μg/mL hygromycin antibiotic selection supplemented into the complete growth medium (GM): Dulbecco’s Modified Eagle Medium (DMEM) supplemented with 10% fetal bovine serum (FBS), 100 IU/mL penicillin and 50 IU/mL streptomycin (1% P/S). Hygromycin antibiotic selection was continued for two days beyond the complete death of negative control wells (non-transfected cells) on day 8, resulting in 10 days of antibiotic selection. Next, the stably transfected adherent YKL-40-HEK293T cells were cultured in GM in a 5% *v*/*v* CO_2_ humidified incubator to produce and secrete the his-tagged YKL-40 protein. After culturing the cells for 48–72 h, the cultured GM was collected, spun at 1000 g for 10 min and stored at −20 °C until protein purification.

### 4.3. YKL-40 Purification

Recombinant human His-tagged YKL-40 protein was purified from the culture medium in a two-step purification process. The first step was purification on a pre-packed 5 mL HisTrap Ni-NTA FPLC column (Cytiva, Marlborough, MA, USA), in accordance with the manufacturer’s instructions, with the following changes: NPI-0 buffer was used for equilibration of the Ni-NTA column, NPI-40 was used as the washing buffer, and NPI-250 was used as the elution buffer (a single step elution from NPI-40 to NPI-250). All buffers and sample solutions were set to pH 8.0. Conditioned GM cell medium was thawed and filtered, its pH was set to 8.0, and it was loaded on the Ni-NTA column using a fast protein liquid chromatography system (FPLC, Pharmacia). The eluted protein fractions were pooled, centrifuged, and dialyzed against a heparin-binding buffer (20 mM phosphate, 50 mM NaCl, pH 7.4) using an Amicon-4 10 kDa filter unit. In the second purification step, the nickel-purified YKL-40 protein was purified on a 5 mL heparin HiTrap column. Heparin-binding buffer was used as the equilibrating, loading, and washing buffer. A heparin elution buffer (20 mM phosphate, 1 M NaCl, pH 7.4) was used to elute the bound YKL-40 protein (single step elution from the heparin-binding buffer to the heparin elution buffer). The heparin-eluted protein fractions were pooled, centrifuged, and dialyzed against MST assay buffer (50 mM phosphate, 50 mM NaCl, pH 7.4). The final protein concentration was calculated using the NanoDrop (Thermo Fisher Scientific, Waltham, MA, USA) spectrometric determination of protein concentration at 280 nm (calculated using MW 40.5 kDa, εmolar 67,840 M^−1^ cm^−1^), and the purity was determined at >90% by SDS-PAGE. To confirm the functional activity of the newly purified recombinant YKL-40 protein, a MST binding affinity experiment was carried out between YKL-40 and a chitin hexamer (A6), with results comparable to published binding values [2,4].

### 4.4. GAG Ligand Selection for In Vitro Studies

**Figure 8:**
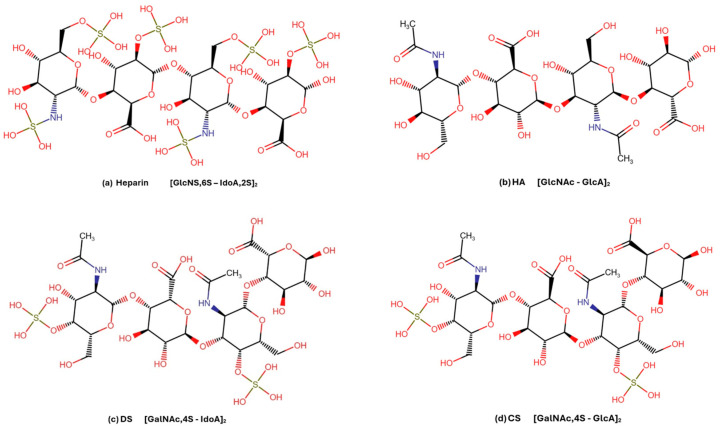
Glycosaminoglycan (GAG) tetrasaccharides used in the molecular docking analysis. The structure of the major repeating disaccharide units of (**a**) Heparin, (**b**) Hyaluronan (HA), (**c**) Dermatan sulfate (DS), and (**d**) Chondroitin sulfate (CS), generated using the RCSB PDB Chemical Sketch Tool (Marvin JS by ChemAxon, version 25.1.0). The tetrasaccharide structures show the repeating disaccharide units of each GAG, their monosaccharide types, sulfation patterns, and stereochemistry.

For the assessment of binding affinity using Microscale Thermophoresis (MST), four GAG ligands were selected: Heparin, HA, DS (CS-B), and CS (a mixture of CS-A and CS-C, with 4S > 6S). Ligand selection was based on the quality of characterization, the defined degree of polymerization (DP), and well-defined disaccharide composition, as specified by the manufacturer (Iduron, Ltd., Cheshire, UK). These criteria ensured a reliable comparison of YKL-40 binding affinities across structurally distinct GAG types (Table 3, Figure 8).

### 4.5. Microscale Thermophoresis (MST)

The binding affinity of various GAG ligands to YKL-40 was determined by His-tag-labeled YKL-40 fluorescence using a MST NT.115 instrument—Blue/Red (NanoTemper Technologies GmbH, Munich, Germany). MST traces were used to determine the binding affinity. All measurements were carried out under temperature-controlled conditions, at 25 °C, using Monolith NT.115 standard capillaries, 40–50% excitation power, MST power at medium to high, and a concentration of 40 nM his-tag-labeled YKL-40. The GAG ligands analyzed were heparin DP6 and DP12, CS DP6, DS DP6, and HA DP6.

A positive control experiment was performed, analyzing YKL-40 with an A6 ligand of known binding affinity (K_d_ 4.0 µM). YKL-40 and GAG-ligand stock solutions were prepared in MST assay buffer: 50 mM phosphate buffer, 50 mM NaCl, 0.05% Tween-20, pH 7.4, and spun for 20 min at 14,500× *g* before measurement.

His-tag YKL-40 fluorescence labeling was performed by following the Monolith His-Tag Labeling Kit RED-tris-NTA 2nd Generation protocol [70]. Initially, Step A—Affinity between His-tag dye and the His-tagged YKL-40 protein was performed and resulted in K_d_ ≈ 10 nM. Based on results from Step A, the step B protein labeling section I was carried out.

Sample preparation was performed by following the MST software instructions provided by the pre-determined Binding-Check and Binding-Affinity assay modules within the MO-control software (MO.Control v1.6). All samples were incubated for at least 20 min before measurement. The GAG concentration range used in each measurement was based on an approximately 50-fold estimated K_d_ with 2-fold serial dilutions, a total of 16 samples in each experiment. Each sample was measured twice, with 2 repetitions (total of 4 measurements). If major aggregation, adsorption, and/or the IF variation were out of a 10% range between measurements, those data points were excluded.

### 4.6. Statistics, Data Fitting, and Graphics

MST raw data was exported from the Monolith instrument and normalized to the lowest ligand concentration. Normalized MST binding curves were fitted against the ligand concentration using nonlinear regression in SigmaPlot 13.0, with the Ligand Binding, one site saturation equation:

(1) $$y=\frac{B_{max}\left|x\right|}{K_{d}+\left|x\right|}$$

where y is the normalized fluorescence signal, |x| is the absolute value of the ligand concentration (μM), B_max_ is the maximum binding capacity (the signal at full receptor saturation), and K_d_ is the dissociation constant.

The fit was calculated with parameter constraints B_max_ > 0 and K_d_ > 0. The K_d_ values were estimated for each measurement (*n* = 4) and presented as K_d_ ± SD. For each ligand, the mean fit of averaged data points between all replicates was also calculated and plotted with standard-error-of-the-mean (SEM) bars.

### 4.7. In Silico Molecular Docking Analysis

A molecular docking analysis was carried out to evaluate the binding interactions between GAG ligands and YKL-40. Two docking software packages were employed for the analysis: AutoDock Vina (version 1.2.7) and Glide (version 2024-4, Schrödinger LLC, New York, NY, USA). AutoDock Vina was used for the first approach blind docking analysis, while Glide was used for refined docking with defined grid-box parameters. To illustrate the final ligand-protein interactions, 2D Ligand-Interaction Diagrams (LIDs) were generated using the Schrödinger’s Maestro interface, with the residues’ cutoff set to 4.0 Å. These 2D LID highlighted the key interacting residues to the corresponding chemical groups of the ligand and type of bonding. Additionally, a docking analysis, including a contact and clash assessment, salt bridge and H-bond analysis, was performed using UCSF Chimera X (version 1.9).

#### 4.7.1. GAG Ligand Construction

The ligands were constructed and energy minimized structures were downloaded using the GAG Builder from Glycam-Web [71] (Complex Carbohydrate Research Center, University of Georgia, Athens, GA, USA; https://glycam.org/gag/#; accessed on 15 May 2025), a tool designed to generate stereochemically, conformationally and structurally accurate GAG structures. This tool applies validated glycosidic linkages and configurations to produce biologically relevant 3D structures optimized for molecular modeling. To comply with AutoDock Vina’s torsion limit of 32 rotatable bonds, GAG sequences were constructed as tetrasaccharides derived from their repetitive disaccharide units (Table 3, Figure 8). This approach focused on capturing the core structural features of each GAG ligand tested in the MST experiments while ensuring compatibility with the torsion limit.

#### 4.7.2. AutoDock Vina Docking

Protein and ligand structures were prepared for Vina docking using AutoDock Tools (ADT, version 1.5.7). Crystal structures of native YKL-40 and its chitin octamer complex were obtained from the Protein Data Bank (PDB IDs: 1NWR and 1HJW). The preparation involved removing all non-standard residues, solvents and water molecules, repairing incomplete side chains, adding polar hydrogen at pH 7.4, and assigning Gasteiger charges. During the docking process, the protein was kept as a rigid structure, while the GAG ligand was allowed full flexibility, based on its rotatable bonds. The docking was performed either as a blind search (grid dimensions: 126 × 126 × 126 Å, covering the whole protein), or centered to the heparin-binding site 143–149 (GRRDKQH), with grid dimensions of 50 × 50 × 50 Å. Docking parameters were set to an exhaustiveness of 32, with 10 node runs, and an energy range of 4 kcal/mol.

#### 4.7.3. Glide Docking

For Glide docking, the protein receptors were dehydrated and prepared by the Protein Preparation Wizard using default settings for H-bond optimization, protonation states, and energy minimization. Following that, docking grids were generated by centering on the chitin- (for PDB IDs: 1NWR and 1HJW) and heparin-binding sites (for PDB ID: 1NWR), with grid dimensions of 80 × 80 × 80 Å. The GAG structures were prepared using LigPrep, to apply the OPLS-2005 force-field for energy minimization, and to optimize the structures and add hydrogen atoms. Furthermore, Epik was used to assign the protonation states at pH 7.4 ± 0.2 and possible tautomers were generated for each molecule by determining their chirality from 3D structures. The docking was performed in SP mode, with flexible ligand sampling, and without applying any constraints. 

## Figures and Tables

**Table 1:** Putative GAG ligands of YKL-40 with structural properties and predicted binding modes. Summary of putative GAG ligands of YKL-40, including their major chemical components and predicted binding interaction (based on known binding domains or relevant surface features supported in the literature).

Ligand	Main Chemical Components	Predicted Binding Site	Binding Likelihood	Justification for Binding
Chitin	GlcNAc (β1→4) GlcNAc	Chitin-binding site. Multiple hydrogen bonds formed and hydrophobic interactions with conserved residues in the chitin-binding groove.	Known ligand	Established experimentally; numerous crystallized structures available [1,2,3,4].
Heparin	IdoA,2S (α1→4) GlcNS (α1→4)	Heparin-binding site (positively charged surface area). Highly sulfated; too negatively charged for tight chitin-binding site fit; interactions via electrostatic interactions and hydrogen bonding.	Known ligand	Binding to positively charged surface area (location not confirmed) due to high negative charge; observed binding in multiple studies; is used in YKL-40 purification processes [4,6]
Hyaluronan	GlcA (β1→3) GlcNAc (β1→4)	Likely chitin-binding site. Structurally similar to chitin; includes GlcNAc, negatively charged; fits chitin groove lined with polar and aromatic residues.	High	Strong hydrogen bonding and possibly some stacking compatibility to chitin-binding site; favored in simulations [7].
Heparan sulfate	GlcA/IdoA (α1→4) GlcNAc/NS	Likely heparin-binding site (positively charged surface area). Similar to heparin but with lower sulfation; structural heterogeneity.	Moderate	Structurally similar to heparin, moderate binding predicted due to lower sulfation [7,8,9].
Chondroitin sulfate	GlcA (β1→3) GalNAc,4S/6S (β1→4)	Likely heparin-binding site (positively charged surface area). Moderate negative charge; sulfate-mediated contacts possible.	Moderate	Plausible ionic- and hydrogen bonding but less favorable fit than heparin due to lower sulfation pattern and lower flexibility; binding supported by modeling and literature evidence [7,49].
Dermatan sulfate	IdoA (β1→3) GalNAc,4S (β1→4)	Likely heparin-binding site (positively charged surface area). More flexible than chondroitin sulfate due to iduronic acid; better fit for sulfate-mediated contacts.	Moderate	Slightly improved binding over chondroitin sulfate due to increased flexibility [7,49].
Keratan sulfate	GlcNAc,6S (β1→3) Gal (β1→4)	Likely heparin-binding site (positively charged surface area); also compatible with chitin-binding site. Lacks uronic acid; fewer possible sulfation sites.	Moderate-Low	Moderate-low likelihood predicted due to lower degree of sulfation and less flexibility; not evaluated in any binding study.

**Table 2:** Binding affinity results for YKL-40 interaction with GAG ligands. Dissociation constant (K_d_) values are represented as mean K_d_ ± standard deviation (SD). The *p*-value reflects the statistical significance of the K_d_ fits of the mean K_d_ from the regression model. Major disaccharide components are shown to reflect the predominant repeating units present in each GAG oligomer.

GAG	Major Disaccharide Units	Mean K_d_ ± SD (μM)	*p*-Value	Notes
Hep DP12	IdoA(2S)-GlcNS(6S)	119 ± 36	<0.0001	High degree of sulfation, includes IdoA
Hep DP6	IdoA(2S)-GlcNS(6S)	234 ± 133	0.0037	Shorter chain, still highly sulfated and contains IdoA
DS DP6	IdoA-GalNAc(4S)	269 ± 30	<0.0001	Lower degree of sulfation than heparin, contains IdoA.
HA DP6	GlcA-GlcNAc	1010 ± 244	<0.0001	No sulfate, no IdoA. Includes GlcNAc (same as chitin).
CS DP6	GlcA-GalNAc(4S > 6S)	Not applicable	0.5530 *	No significant binding. Lower degree of sulfation than heparin; does not contain IdoA.

* Fit not significant (*p* > 0.05). Data shown in Supplemental Figure S2.

**Table 3:** An overview of the glycosaminoglycan (GAG) ligands from Iduron Ltd. used in the binding affinity experiments. This table lists the catalog numbers, general chemical formulas, and minor quantities of GAGs purchased from Iduron Ltd. These ligands were used in the Microscale Thermophoresis (MST) experiments to evaluate the binding interactions. All the information was provided in the manufacturer’s specifications.

GAG (Cat. No.)	General Formula (*n* = 2 for DP6, and *n* = 5 for DP12)	Minor Quantities
Heparin (HO06 and HO12)	UA,2S − (GlcNS,6S − IdoA,2S)_n_ − GlcNS,6S	di-, mono-, and non-sulfated units
Dermatan sulfate (DSO06)	∆UA − (GalNAc,4S − IdoA)_n_ − GalNAc,4S	6-sulfated and 2,4 di-sulfated units (5% and 7%, respectively)
Chondroitin sulfate (CSO06)	∆UA − (GalNAc,4S * > 6S − GlcA)_n_ − GalNAc,4S > 6S	GlcA,2S-GalNAc,6S (5%)
Hyaluronan (HA06)	∆HexAβ1,3 − (GlcNAcβ1,4 GlcAβ1,3)_n_ – GlcNAc	none

* The major compound is a mixture of CS-A, CS-C (4S > 6S), and minor quantities of CS-D.

## Data Availability

The original data presented in the study are included in the article and Supplementary Material; further inquiries can be directed to the corresponding author.

## Supplementary Materials

The following supporting information can be downloaded at https://www.mdpi.com/article/10.3390/md23100379/s1, Figure S1: Binding affinity curves between YKL-40 and four GAG ligands (heparin, dermatan sulfate (DS), and hyaluronan (HA)), measured by Microscale Thermophoresis (MST).; Figure S2: Binding affinity curves between YKL-40 and chondroitin sulfate (CS) DP6, measured by Microscale Thermophoresis (MST); Table S1: Docking results of various glycosaminoglycan (GAG) ligands with YKL-40, showing Docking scores, Glide emodel values, and Glide scores from Schrödinger.

## Abbreviations

The following abbreviations are used in this manuscript:

GAG	Glycosaminoglycans
MST	Microscale Thermophoresis
Hep	Heparin
HS	Heparan sulfate
DS	Dermatan sulfate
CS	Chondroitin sulfate
KS	Keratan sulfate
HA	Hyaluronan
GlcA	D-glucuronic acid
IdoA	L-iduronic acid
IdoA,2S	2-sulfated iduronic acid
GlcNS	N-sulfated D-glucuronic acid
GlcNS,6S	6-O-sulfated N-sulfated D-glucuronic acid
GlcNAc	N-acetyl D-glucosamine
GalNAc	N-acetyl D-galactosamine
GalNAc,4S	4-O-sulfated N-acetyl D-galactosamine
DP	Degree of polarization
ChOS	Chitooligosaccharides
FAK	Focal adhesion kinase
MAPK	Mitogen-activated protein kinase
ERK	Extracellular signal-regulated kinase
PI3K	Phosphoinositide 3-kinase
AKT	Protein kinase B
ECM	Extracellular Matrix
PGs	Proteoglycans
HEK293T	Human embryonic kidney cells 293T
LID	Ligand-Interacting Diagrams
