# Supplementary material for: Characterization of YKL-40 Binding to Extracellular Matrix Glycosaminoglycans
Source: Mar Drugs. 2025 Sep 26;23(10):379. doi: 10.3390/md23100379 (PMC12565052; doi:10.3390/md23100379)
Supplement: Supplementary file 1 [file marinedrugs-23-00379-s001.zip › marinedrugs-3865190-supplementary.pdf]

Supplementary Materials

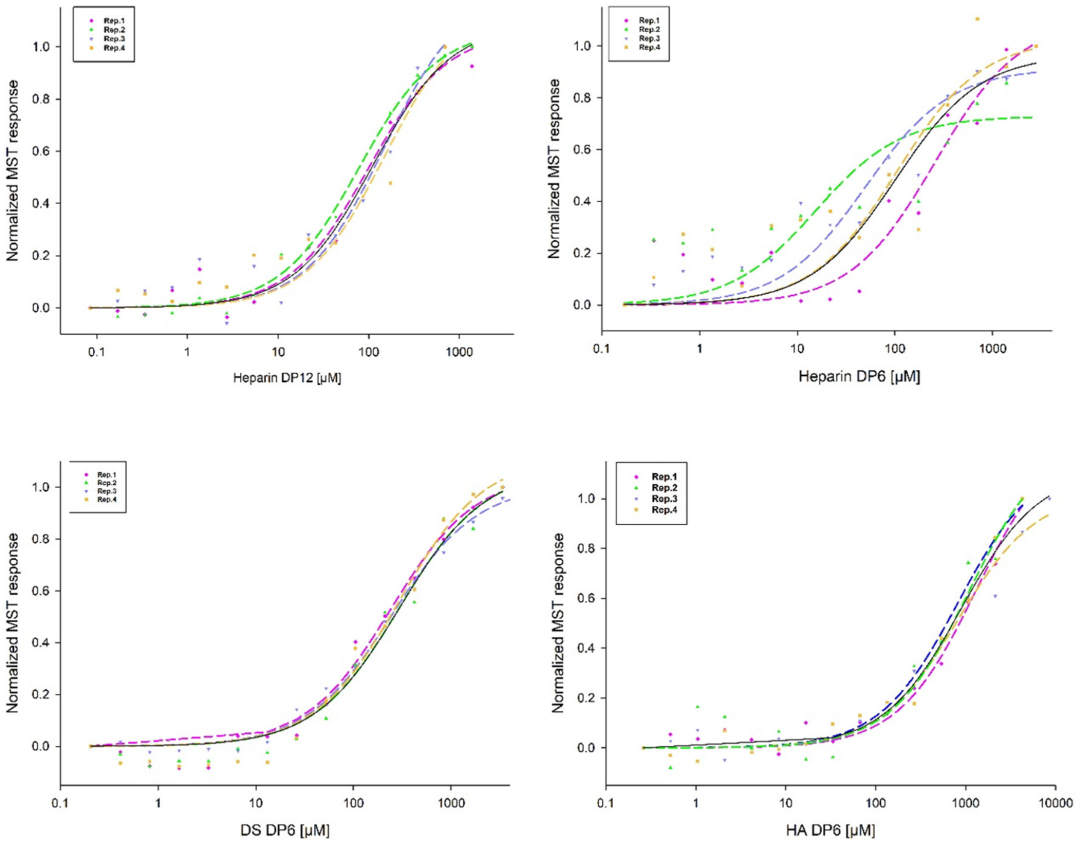

Supplemental Figure S1. Binding affinity curves between YKL-40 and four GAG ligands (heparin, dermatan sulfate (DS), and hyaluronan (HA)), measured by Microscale Thermophoresis (MST). Analysis of the dissociation constant (K_d) was performed using his-tag labelled YKL-40 protein and heparin DP12, heparin DP6, DS DP6, and HA DP6 ligands. MST binding curves of four individual measurements (rep.1-4) were fitted using nonlinear regression in SigmaPlot 13.0, with the Ligand Binding, one site saturation equation: f = Bmax*abs(x)/(Kd + abs(x)), with parameter constraints Bmax >0, and Kd >0. The MST dose-response binding curves are displayed in pink (rep.1), green (rep.2), blue (rep.3), and orange (rep.4). The mean fit of averaged data points between all replicates is displayed as a thin black line.

Supplemental Figure S2 shows the normalized MST response for CS DP6 from four individual measurements (rep.1-4) and the mean fit of averaged data points between all replicates (to the left), and an example of the low amplitude MST trace (to the right). None of the four replicates showed statistically significant binding, and thus, calculating the mean K_d is not appropriate.

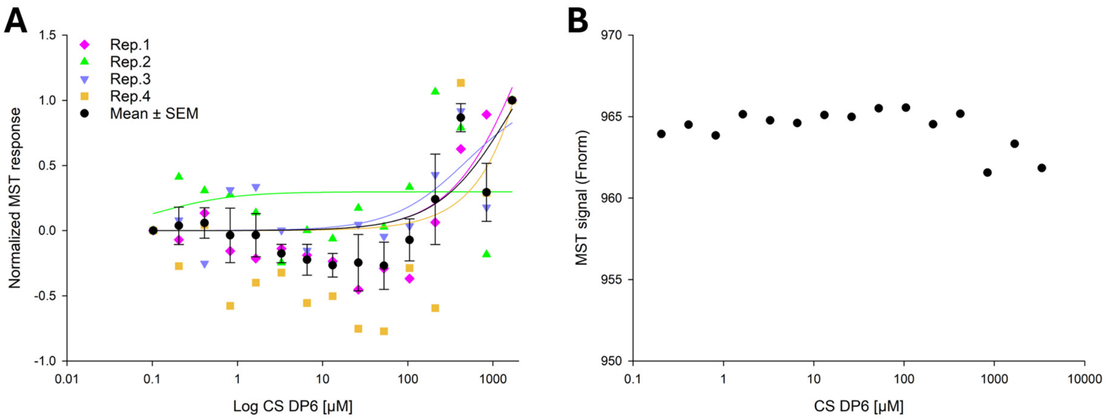

Supplemental Figure S2. Binding affinity curves between YKL-40 and chondroitin sulfate (CS) DP6, measured by Microscale Thermophoresis (MST). Analysis of the dissociation constant (K_d) was performed using his-tag labelled YKL-40 protein and CS DP6 ligand. A) Normalized MST binding curves of four individual measurements (rep.1-4) fitted using nonlinear regression in SigmaPlot 13.0, with the Ligand Binding, one site saturation equation: f = Bmax*abs(x)/(Kd + abs(x)), with parameter constraints Bmax >0, and Kd >0. The MST dose-response binding curves are displayed in pink (rep.1), green (rep.2), blue (rep.3), and orange (rep.4). The mean fit of averaged data points between all replicates is displayed as a thin black line. B) An example of the low MST trace data (from rep.4), which limits the normalization of the data and reliability of binding curve fitting.

Supplemental Table S1. Docking results of various glycosaminoglycan (GAG) ligands with YKL-40, showing Docking scores, Glide emodel values, and Glide scores from Schrödinger. For Docking score and Glide score, lower (more negative) values indicate more favorable predicted binding. Glide emodel is a pose-ranking score combining binding energy and ligand strain (more negative indicates more favorable docking poses).

Sample name	Docking score (kcal/mol)	Glide emodel (kcal/mol)	Glide score (kcal/mol)
Hep	-4.86	-67.08	-4.86
DS_Pose 1	-5.906	-77.056	-6.343
DS_Pose_2	-4.278	-59.389	-4.872
CS_Pose_1	-3.581	-63.742	-4.019
CS_Pose_2	-4.872	-60.992	-5.31
Chit_1HJW_HA	-8.713	-115.86	-8.713
Chit_1NWR_HA	-6.933	-94.772	-6.933
GRRDKQH_HA_Pose_1	-5.937	-75.73	-5.937
GRRDKQH_HA_Pose_2	-4.349	-47.454	-4.349
